# Role of peroxiredoxin2 downregulation in recurrent miscarriage through regulation of trophoblast proliferation and apoptosis

**DOI:** 10.1038/cddis.2017.301

**Published:** 2017-06-29

**Authors:** Fan Wu, Fuju Tian, Weihong Zeng, Xiaorui Liu, Jianxia Fan, Yi Lin, Yan Zhang

**Affiliations:** 1 Institute of Embryo-Fetal Original Adult Disease Affiliated to Shanghai Jiao Tong University School of Medicine, the International Peace Maternity & Child Health Hospital, Shanghai Jiao Tong University School of Medicine, Shanghai, China; 2 Department of Obstetrics and Gynecology, Renmin Hospital of Wuhan University, Wuhan, China

## Abstract

Peroxiredoxin (Prdx) 2 is an antioxidant protein that utilizes its redox-sensitive cysteine groups to reduce hydrogen peroxide molecules and protect cells against oxidative damage from reactive oxygen species (ROS). However, its function in trophoblasts at the maternal–fetal interface has not been clarified yet. In this study, significantly lower Prdx2 expression was found in the first-trimester villous cytotrophoblasts of patients with recurrent miscarriage (RM) than in cytotrophoblasts from healthy controls. Further, Prdx2 knockdown inhibited proliferation and increased apoptosis of trophoblast cells. The reason for this may be an increase in the level of cellular ROS after knockdown of Prdx2, which may subsequently lead to an increase in the expression of phosphorylated p53 (p-p53) and p38-MAPK/p21. Prdx2 knockdown also impaired the fusion of BeWo cells induced by forskolin. Bioinformatics analysis identified a c-Myc-binding site in the Prdx2 promoter region, and chromatin immunoprecipitation verified that c-Myc directly bound to a site in this locus. Suppression and overexpression of c-Myc resulted in reduction and increase of Prdx2 expression respectively. Furthermore, we demonstrated that c-Myc was downregulated in the first-trimester cytotrophoblasts of patients with RM, and its downregulation is also related with inhibited cell proliferation, increased apoptosis, as well as upregulated p21 expression and p-p53/p53 ratio. Our findings indicate that Prdx2 might have an important role in the regulation of trophoblast proliferation and apoptosis during early pregnancy, and that its expression is mediated by c-Myc. Thus, these two proteins may be involved in the pathogenesis of RM and may represent potential therapeutic targets.

During pregnancy, the placenta is in an active metabolic state and continually produces reactive oxygen species (ROS).^1^ Under normal conditions, oxidation and reduction factors are in a state of delicate balance, and ROS act as secondary messengers in trophoblast proliferation and differentiation.^1, 2^ However, if there is an imbalance in the redox state, spontaneous abortion, preeclampsia or intrauterine growth restriction could occur.^1^ Although ROS are necessary for cell growth signaling, high levels of ROS are associated with checkpoint responses that induce a transient or long-term pause in the cell cycle.^3, 4, 5^ The involved proteins include ataxia-telangiectasia mutated kinase (ATM), p53 and p21.^4, 5^ It has been reported that an increase in the ROS level could disrupt mitogenic signaling in the G0 to G1 phase and lead to cell cycle arrest in the G0 phase.^5^

Peroxiredoxins (Prdxs) are a family of efficient ROS scavengers that maintain the cellular reducing milieu using the thiol groups of their cysteine residues to eliminate hydrogen peroxide and other oxidants.^6^ According to the ‘floodgate’ hypothesis of Wood *et al.*,^6^ 2-Cys Prdxs are abundant and ubiquitous in cells and maintain low levels of hydrogen peroxide; however, high levels of peroxides can lead to over-oxidation and subsequently inactivate Prdxs, which allows ROS to accumulate and thus trigger redox-dependent signaling.^6^ The signaling events that follow are very likely to lead to apoptosis.^6^ Other studies have also supported this hypothesis and indicate that Prdxs have a significant role in resistance against pro-oxidation signals.^7^ Further, Phalen *et al.* provided an additional hypothesis, according to which Prdxs may interpret and warn cells of perturbations in oxidant metabolism by using post-translational modifications such as Prdx-SO_2_H and thereby contribute to cell cycle arrest.^8^ With regard to Prdx2 in particular, research has demonstrated its efficiency for scavenging hydrogen peroxide molecules, protecting cells from oxidative stress and prolonging the life span of cells.^9, 10^ Several types of human carcinomas show elevated levels of Prdx2, which is related with their strong proliferation rate and capacity for tolerating chemotherapy.^11, 12^ Prdx2 also regulates the metastasis of carcinomas by negatively regulating Src/ERK activation.^13^ From all these findings, it can be concluded that Prdx2 not only ameliorates ROS-related damage, but also acts as an ROS signal receptor and transmitter.^14, 15^

Recurrent miscarriage (RM) occurs in 1–3% of women of reproductive age, and it is defined as three or more consecutive spontaneous abortions before 20 weeks of gestation.^16^ Enhanced ROS activity along with impairment in the antioxidant system has been implicated in the pathogenesis of RM.^17^ Moreover, apoptosis is much more evident in the human conceptus in the first-trimester in cases of RM than in normal pregnancy.^18^ The relationship between Prdx2 and RM, as well as the upstream regulation of Prdx2, has not been sufficiently explored. One study showed that Prdx3 and Prdx4 may have an important role in implantation and production of autoimmune antibodies against them may be related with miscarriages.^19^ Another study showed that FKBP52-Prdx6 signaling is useful for successful implantation.^20^ Further, our previous research demonstrated that Prdx2 expression was significantly downregulated in uterine lymphocytes isolated from NOD mice with impaired fertility, and that Prdx2 inhibition increased the cytotoxicity of uNK cells and thus increased the percentage of embryo loss in CBA/J × DBA/2J mice.^21, 22^ However, no studies have yet investigated the role of Prdx2 in trophoblasts of RM, which makes the present study the first of its kind. In this study, we investigated the role of Prdx2 in RM. Our findings indicated that c-Myc regulation of Prdx2 might have an important role in trophoblast proliferation and apoptosis, and that this may be involved in the pathogenesis of RM.

## Results

### Downregulation of Prdx2 in cytotrophoblasts from patients with RM

**Figure 1:**
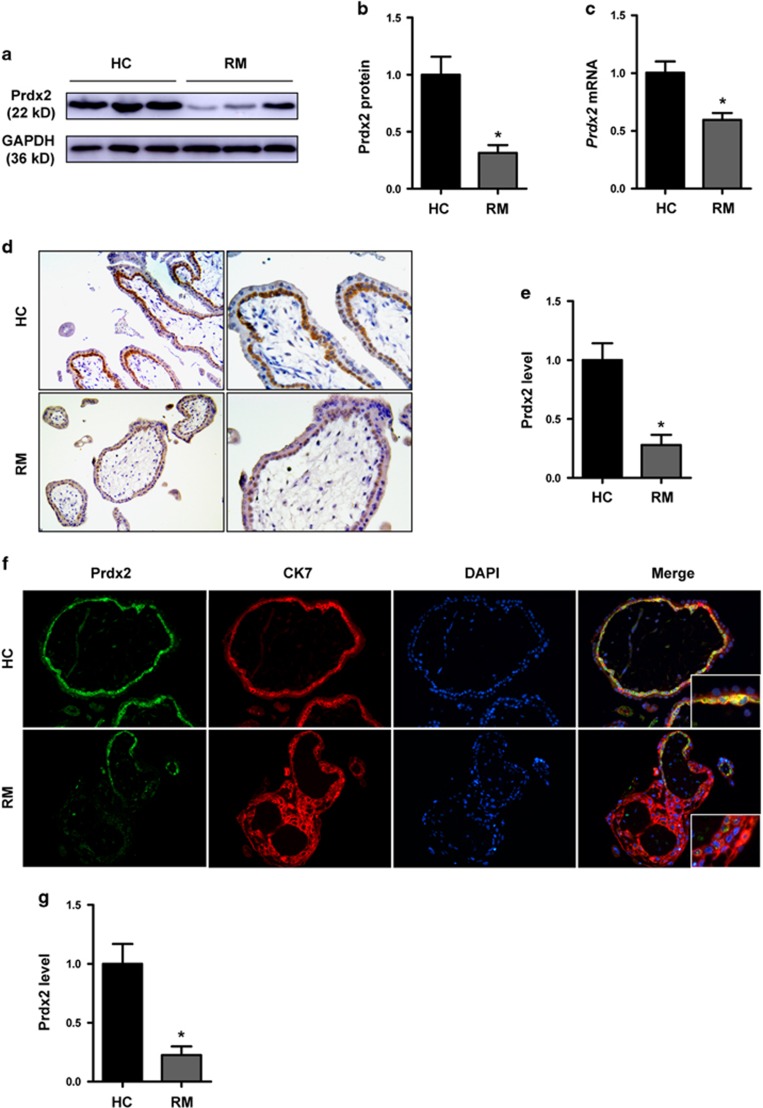
Peroxiredoxin (Prdx) 2 is downregulated in first-trimester villous cytotrophoblasts in patients with recurrent miscarriage (RM). (**a**–**c**) Prdx2 levels in first-trimester villous tissues from patients with RM and healthy controls (HCs) were determined by western blot and quantitative RT-PCR analysis. The gray value of the western blot bands was determined by Gel-Pro Analyzer 4.0. (**d**,**e**) Immunohistochemical assay was performed on sections of first-trimester villi developed with the horseradish peroxidase-conjugated streptavidin–biotin complex with the chromogen 3,3-diaminobenzidine and counterstained with hematoxylin. Original magnification: 100 × (left), 200 × (right). (**f**,**g**) Double immunofluorescence staining for trophoblast marker cytokeratin 7 (CK7) with Prdx2 was performed in first-trimester human villi from patients with RM and HCs. Green fluorescent signals represent Prdx2 antibodies, red signals represent CK7 antibodies and the blue fluorescent signals represent nuclei. Images were obtained using a fluorescence microscope (Leica DMi8). Original magnification: 200 ×. Positive cells from both the immunohistochemical and immunofluorescence assays were quantified using ImagePro-plus 6.0. **b** and **c**: *n*=11 for each group; **e** and **g**: *n*=12 for each group. Data are expressed as the mean±S.E.M. *P<0.05 *versus* HCs. GAPDH, glyceraldehyde-3-phosphate dehydrogenase; DAPI, 4′,6-diamidino-2-phenylindole

First-trimester chorionic villous tissues were collected to investigate the role of Prdx2 in the pathogenesis of RM. The results of quantitative RT-PCR and western blot analysis showed that Prdx2 protein and mRNA expression was significantly downregulated in the villous tissue of patients with RM compared with healthy controls (HCs) (Figures 1a–c). To further investigate the localization and expression of Prdx2, immunohistochemical (IHC) and immunofluorescence (IF) analyses of paraffin-embedded first-trimester villous tissue were performed. In IHC analyses, much stronger expression of Prdx2 was observed in villous tissue from HCs compared with RM group and the Prdx2-positive cells were mainly found in the cytotrophoblast layer (Figures 1d and e). The trophoblastic cell columns and extravillous trophoblast cells also express Prdx2 (Supplementary Figure 1a and b). In IF analyses, double staining for trophoblast marker cytokeratin 7 (CK7) with Prdx2 was performed. Higher percentage of Prdx2^+^/CK7^+^ cells and stronger Prdx2 staining signal was observed in the villi of HC group compared with that of the RM group (Figures 1f and g). Thus, Prdx2 is downregulated in cytotrophoblasts from patients with RM.

### Inhibition of trophoblast proliferation and induction of trophoblast apoptosis by increasing ROS in Prdx2 knockdown cells

**Figure 2:**
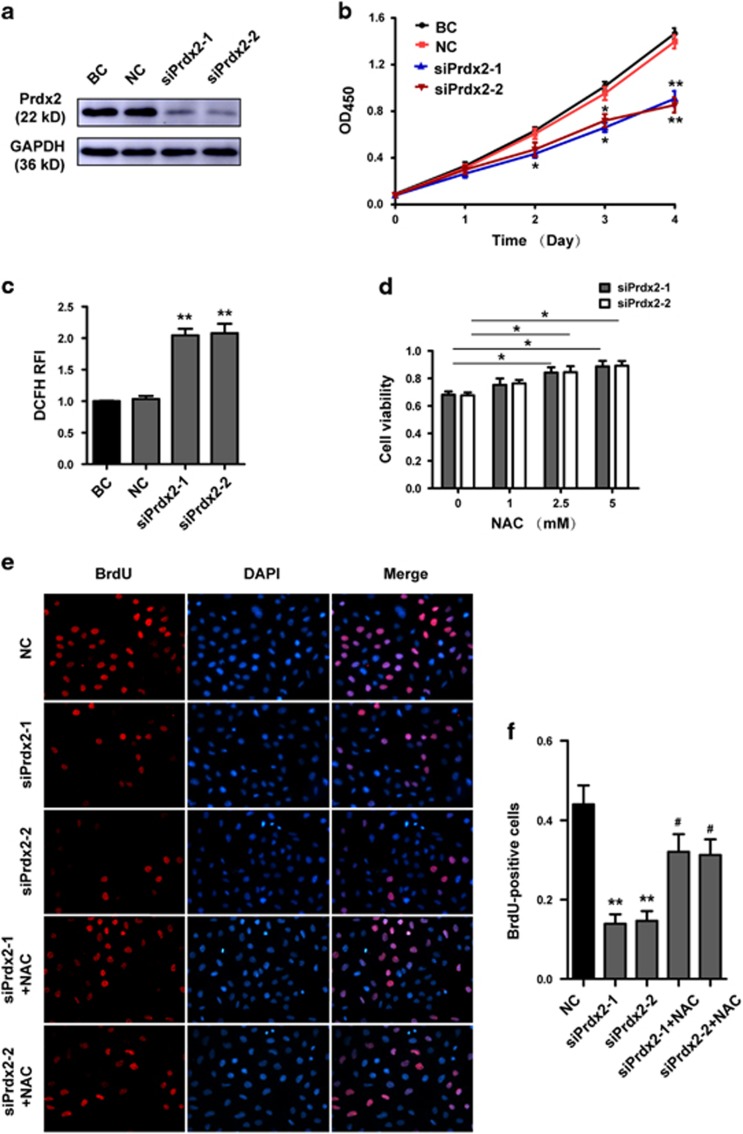

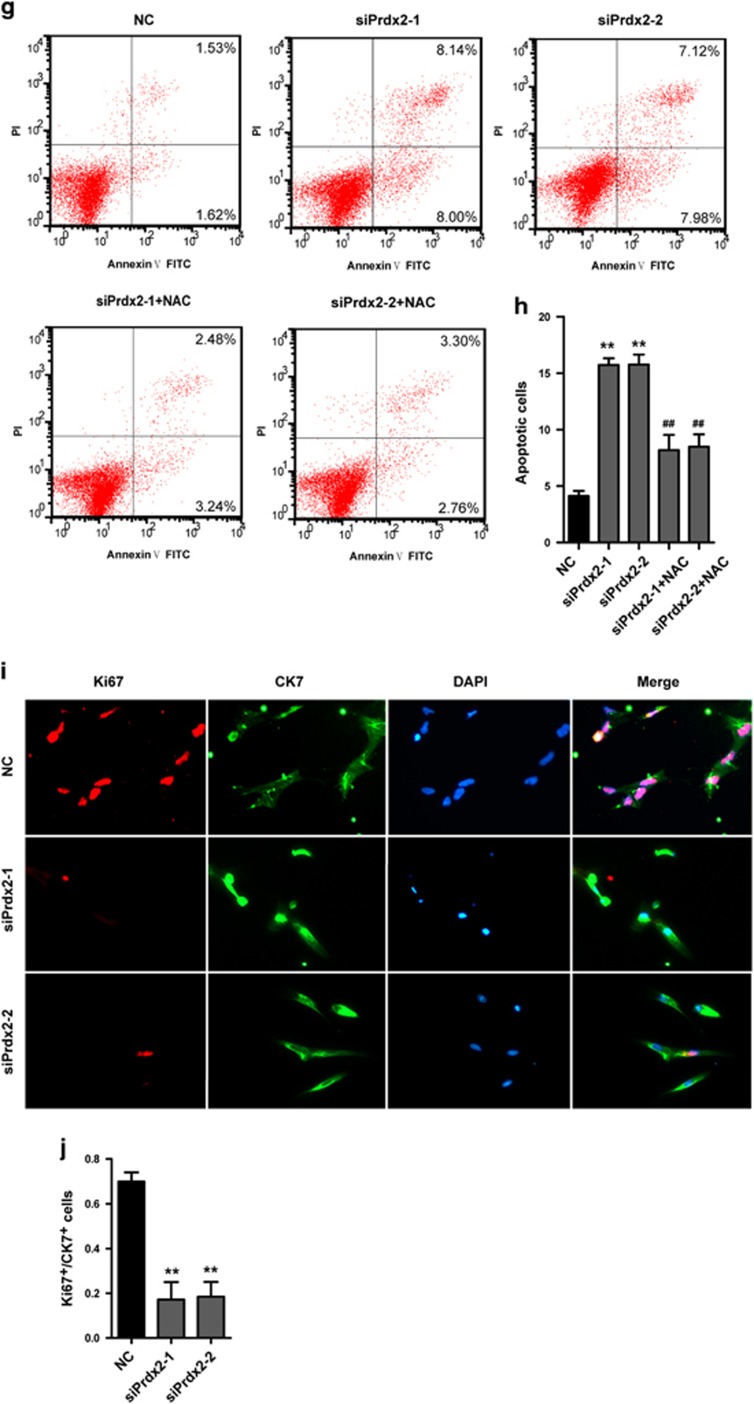
Downregulation of Prdx2 inhibits trophoblast proliferation and increases the rate of apoptosis by inducing endogenous ROS. (**a**) HTR8 cells were transfected with negative control siRNA and siPrdx2. Prdx2 expression levels were analyzed by western blotting after 48 h of transfection. (**b**) Cell proliferation was analyzed by the CCK8 assay after transfection with siPrdx2, and the siPrdx2-transfected cells were compared with the blank control (BC) and negative control (NC) cells. (**c**) ROS accumulation in HTR8 cells was detected using the 2′,7′-dichlorofluorescin diacetate (DCFH-DA) probe. Relative fluorescence intensity (RFI) indicated the intracellular ROS level. (**d**) The viability of siPrdx2-transfected cells treated with different concentrations of the antioxidant NAC was determined by the CCK8 assay. (**e**,**f**) HTR8 cells were treated with control siRNA, siPrdx2 and siPrdx2 in combination with 5 mM NAC. DNA synthesis in the HTR8 cells was determined using 5-bromo-2′-deoxyuridine (BrdU) labeling. Red fluorescent signals indicate BrdU-positive cells, and blue fluorescent signals indicate nuclei. Original magnification: 200 ×. (**g**,**h**) The apoptosis rate of HTR8 cells was determined by flow cytometry analysis after cells were treated with control siRNA, siPrdx2 and siPrdx2 in combination with 5 mm NAC. (**i**,**j**) Whole mount immunofluorescent assay was used to analyze placental villi proliferation after the explants were transfected with control siRNA and siPrdx2. Red fluorescent signals indicate Ki67, green fluorescent signals indicate CK7 and the blue fluorescent signals represent nuclei. The number of Ki67^+^/CK7^+^ cells was calculated, and normalized to the number of total CK7^+^ cells. *n*=11. Original magnification: 200 ×. Data are expressed as the mean±S.E.M. *P<0.05 *versus* BC and NC, **P<0.001 *versus* BC and NC, ^#^*P*<0.05 *versus* siPrdx2-1 or siPrdx2-2, ^##^*P*<0.001 *versus* siPrdx2-1 or siPrdx2-2

We next investigated the effect of Prdx2 on the proliferation and apoptosis of trophoblasts by using the first-trimester human extravillous cytotrophoblast-derived cell line HTR8/SVneo (HTR8). HTR8 cells were transfected with control siRNA and siRNA against Prdx2 (siPrdx2). Prdx2 expression was markedly decreased after transfection with siPrdx2 (Figure 2a). The cell counting kit-8 (CCK8) assay showed that Prdx2 knockdown decreased HTR8 cell proliferation (Figure 2b). We examined endogenous production of ROS in siPrdx2-transfected HTR8 cells. Endogenous ROS was measured with the 2′,7′-dichlorofluorescin diacetate (DCFH-DA) probe. The fluorescence intensity of the siPrdx2-transfected cells was about twice as high as that of the control cells (Figure 2c). We further evaluated cell viability in siPrdx2-transfected cells treated with different concentrations of the antioxidant *N*-acetyl-l-cysteine (NAC). After treatment with NAC (0–5 mM) for 24 h, the CCK8 assay showed that the percentage of viable siPrdx2-transfected cells was higher than the percentage of viable untreated cells (Figure 2d).

The thymidine analog 5-bromo-2′-deoxyuridine (BrdU) can incorporate into cellular DNA during the S phase of the cell cycle, which was used to assess cell proliferation. Knockdown of Prdx2 obviously decreased the proportion of BrdU-labeled cells, which indicated the downregulation of cell proliferation. When siPrdx2-transfected cells were treated with 5 mM antioxidant NAC, the proportion of BrdU-labeled cells increased (Figures 2e and f). We also analyzed cell apoptosis after Prdx2 knockdown by flow cytometry after annexin-V/propidium iodide staining. The results showed that Prdx2 knockdown not only reduced the cell proliferation rate but also increased the cell apoptosis rate. When we treated siPrdx2-transfected cells with 5 mM NAC, the proportion of apoptosis was reduced (Figures 2g and h). These results indicated that Prdx2 could inhibit cell proliferation and induce cell apoptosis through increasing ROS.

To further confirm the role of Prdx2 in trophoblast proliferation *in vivo*, explants from first-trimester healthy villi were cultured on Matrigel-coated plates and transfected with control siRNA or siPrdx2. After 72 h of culture, double IF staining for trophoblast marker CK7 with cell proliferation marker Ki67 was performed. Knockdown of Prdx2 repressed the proliferation of placenta villi as a decreased percentage of Ki67^+^/CK7^+^ cells was shown in siPrdx2-transfected trophoblast cells (Figures 2i and j).

### Downregulated Ki67 and increased apoptosis in villous tissues from patients with RM

**Figure 3:**
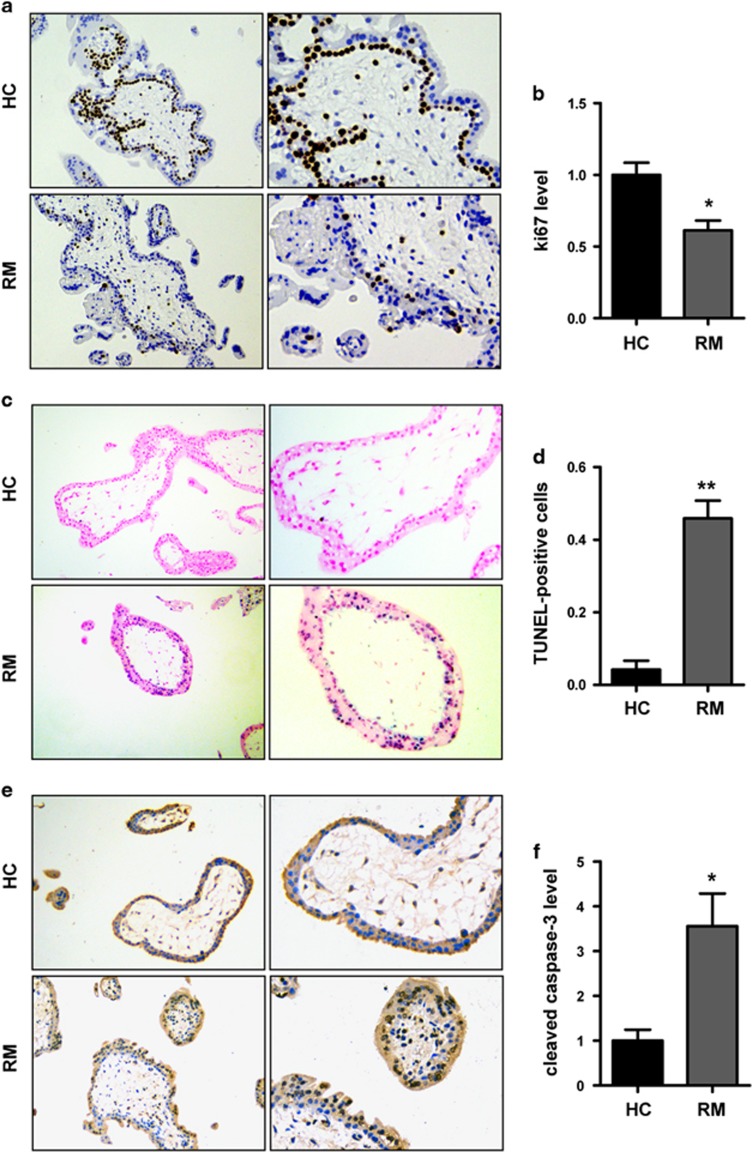
Trophoblast proliferation is downregulated and the rate of apoptosis increases in villous tissues from patients with RM. (**a**,**b**) Villous sections from HCs and patients with RM were subjected to immunohistochemical staining using Ki67 antibodies, developed by horseradish peroxidase-conjugated streptavidin–biotin complex and the chromogen 3,3-diaminobenzidine counterstained with hematoxylin. Cells that were positively stained were quantified using ImagePro-plus 6.0. *n*=12 for each group. Original magnification: 100 × (left), 200 × (right). (**c**,**d**) *In situ* cell death in the first-trimester maternal villi of patients with RM and HCs was analyzed with TUNEL staining by detecting the incorporated fluorescein-dUTP with an alkaline phosphatase-conjugated anti-fluorescein antibody with the chromogen 5-bromo-4-chloro-3-indolyl phosphate/tetranitroblue tetrazolium chloride (BCIP/NBT) and counterstained with nuclear fast red. *n*=11 for each group. Original magnification: 100 × (left), 200 × (right). (**e**,**f**) Villous sections from HCs and RM were subjected to immunohistochemical staining using cleaved caspase-3 antibodies, developed by horseradish peroxidase-conjugated streptavidin–biotin complex and the chromogen 3,3-diaminobenzidine counterstained with hematoxylin. Cells that were positively stained were quantified using ImagePro-plus 6.0. *n*=8 for each group. Original magnification: 100 × (left), 200 × (right). Data are expressed as the mean±S.E.M. *P<0.05 *versus* HCs, **P<0.001 *versus* HCs. TUNEL, TdT-mediated dUTP Nick-End Labeling

We verified the *in vitro* findings above in the villous tissue samples from RM patients and HCs. IHC assay showed that the expression of Ki67 was decreased in the villi of patients with RM, indicating an attenuated trophoblast proliferation in RM (Figures 3a and b). Detection of DNA strand breaks in apoptotic cells was performed with the TUNEL kit. The results showed that in the villi of patients with RM, the percentage of cells undergoing apoptosis is higher than that in HCs (Figures 3c and d). The expression of cleaved caspase-3 was also detected by IHC in placenta tissues and an increased cleaved caspase-3 level was shown in the villi of patients with RM (Figures 3e and f).

### Impairment of BeWo cell fusion after Prdx2 knockdown

**Figure 4:**
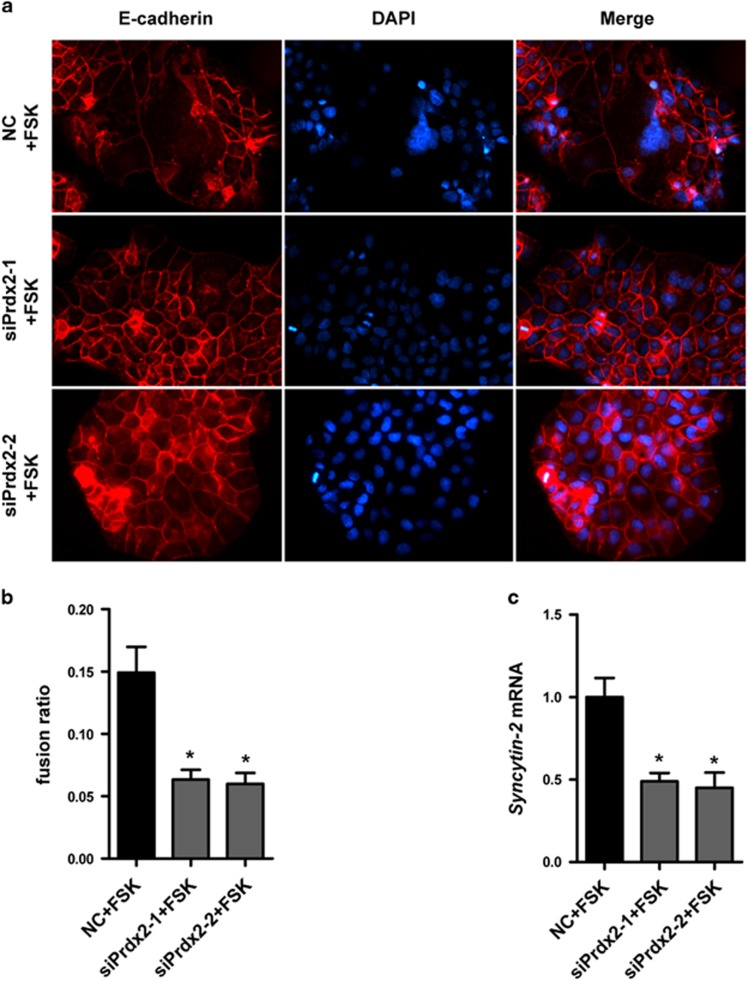
Forskolin (FSK)-induced BeWo cell fusion is reduced after Prdx2 knockdown. (**a**,**b**) BeWo cells were transfected with control siRNA or siPrdx2 and then treated with FSK. Cells were subjected to immunofluorescence staining with E-cadherin antibody (red) and counterstained with DAPI (blue). Original magnification: 200 ×. The percentage of nuclei in syncytial cells was calculated. (**c**) Quantitative RT-PCR was used to analyze *Syncytin-2* expression in FSK-treated BeWo cells that have been transfected with control siRNA or siPrdx2. Data are expressed as the mean±S.E.M. *P<0.05 *versus* NC+FSK

Forskolin (FSK) treated BeWo cells can fuse to form multinucleated cells and thus BeWo cells are often used as a model to study syncytiotrophoblast formation. BeWo cells were transfected with control siRNA or siPrdx2 for 24 h and subsequently treated with 50 *μ*M FSK (Sigma-Aldrich) for 48 h. The fusion ratio of BeWo can be indicated by E-cadherin rearrangement as the E-cadherin IF staining at the intracellular boundaries will disappear after cell fusion. We observed more multinucleated cells with disappeared E-cadherin staining at cell borders in control group. However, lower cell fusion ratio was seen in siPrdx2-transfected BeWo cells (Figures 4a and b). We utilized quantitative RT-PCR to analyze *Syncytin-2* expression, which is a kind of human endogenous retrovirus envelope proteins that have an important role in trophoblast fusion.^23^ The expression of *Syncytin-2* was downregulated in siPrdx2-transfected cells compared with control siRNA transfected cells after FSK treatment (Figure 4c). Above all, Prdx2 knockdown may impair the fusion of trophoblast cells and damage the process of syncytialization.

### Prdx2 knockdown-induced expression of phosphorylated p53 and p38-MAPK/p21

**Figure 5:**
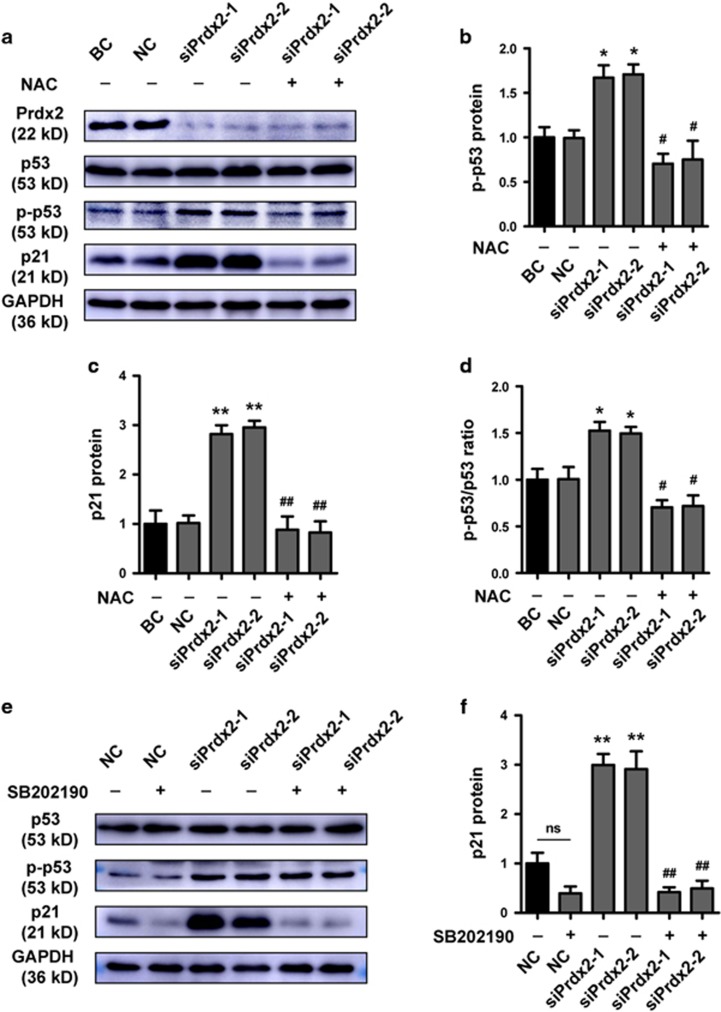

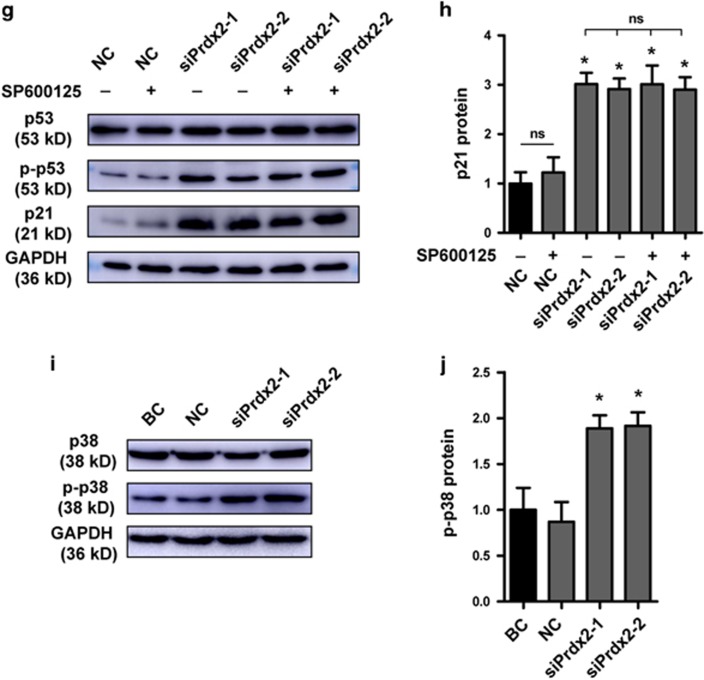
Prdx2 knockdown induces the expression of phosphorylated p53 (p-p53) and p38-MAPK/p21 through endogenous ROS production. (**a**–**d**) The effect of Prdx2 knockdown on the protein level of p53, p-p53 and p21 was analyzed by western blot analysis. NAC was added at a concentration of 5 mm for 24 h in siPrdx2-transfected HTR8 cells, and variations in the p53, p-p53 and p21 level were detected by western blotting analysis. (**e**,**f**) Control and siPrdx2-transfected HTR8 cells were treated with p38-MAPK inhibitor SB202190 at a concentration of 10 *μ*M (+) or vehicle DMSO (−) for 24 h. The expression of p53, p-p53 and p21 were analyzed by western blot. (**g**,**h**) Control and siPrdx2-transfected HTR8 cells were treated with JNK inhibitor SP600125 at a concentration of 10 *μ*M (+) or vehicle DMSO (−) for 24 h. The expression of p53, p-p53 and p21 were analyzed by western blot. (**i**,**j**) The expression of p38 and p-p38 were analyzed by western blot in control and siPrdx2-transfected HTR8 cells. The gray value of all protein bands was determined using Gel-Pro Analyzer 4.0. ns: no significance, *P<0.05 *versus* BC and NC, **P<0.001 *versus* BC and NC, ^#^*P*<0.05 *versus* siPrdx2-1 or siPrdx2-2, ^##^*P*<0.001 *versus* siPrdx2-1 or siPrdx2-2. NAC, *N*-acetyl-l-cysteine; ROS, reactive oxygen species

We investigated the mechanism underlying the observed effects of Prdx2 knockdown on the proliferation and apoptosis of villous and HTR8 cells. With the downregulation of Prdx2 expression, the expression of p21 increased. Although the level of total p53 was not influenced, phosphorylated p53 (p-p53) was upregulated. When 5 mM of NAC was added, p-p53 and p21 expression was almost downregulated to the control level (Figures 5a–d). These results indicate that cell cycle arrest and apoptosis were induced by ROS, and that this effect could be associated with increased p-p53/p53 ratio and p21 expression. Further, the inhibitors SB202190 (Sigma-Aldrich) and SP600125 (Sigma-Aldrich) were used to block p38 mitogen-activated protein kinase (p38-MAPK) and Jun N-terminal kinase (JNK) respectively in control and siPrdx2-transfected HTR8 cells. Western blot analysis showed that SB202190, not SP600125, could block the increase of p21 after Prdx2 knockdown with no influence on p-p53 or p53 (Figures 5e–h). We verified the p38 phosphorylation activation (p-p38) after Prdx2 knockdown by western blot analysis (Figures 5i and j).

### Upregulation of p-p53 and p21 in villous tissues from patients with RM

**Figure 6:**
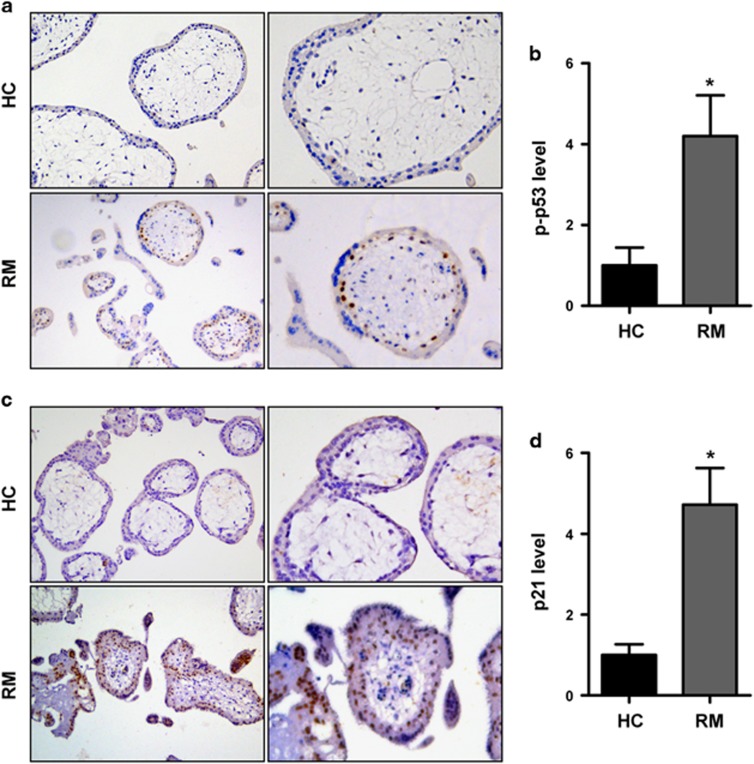
p-p53 and p21 are upregulated in villous tissues from patients with RM. Villous sections from HCs and patients with RM were subjected to immunohistochemical staining using p-p53 (**a**,**b**); b: *n*=12 for HCs, *n*=10 for the RM group) or p21 antibodies (**c**,**d**); d: *n*=11 for HCs, *n*=12 for the RM group), developed by horseradish peroxidase-conjugated streptavidin–biotin complex and the chromogen 3,3-diaminobenzidine counterstained with hematoxylin. Cells that were positively stained were quantified using ImagePro-plus 6.0. Original magnification: 100 × (left), 200 × (right). Data are expressed as the mean±S.E.M. *P<0.05 *versus* HCs

We measured the level of p-p53 and p21 in the villous tissue of patients with RM and HCs with the IHC assay. The results showed an increase in p-p53 and p21 expression in RM patients (Figures 6a–d). The p53 level was also upregulated in patients with RM (Supplementary Figure 2a and b).

### Regulation of Prdx2 by c-Myc

**Figure 7:**
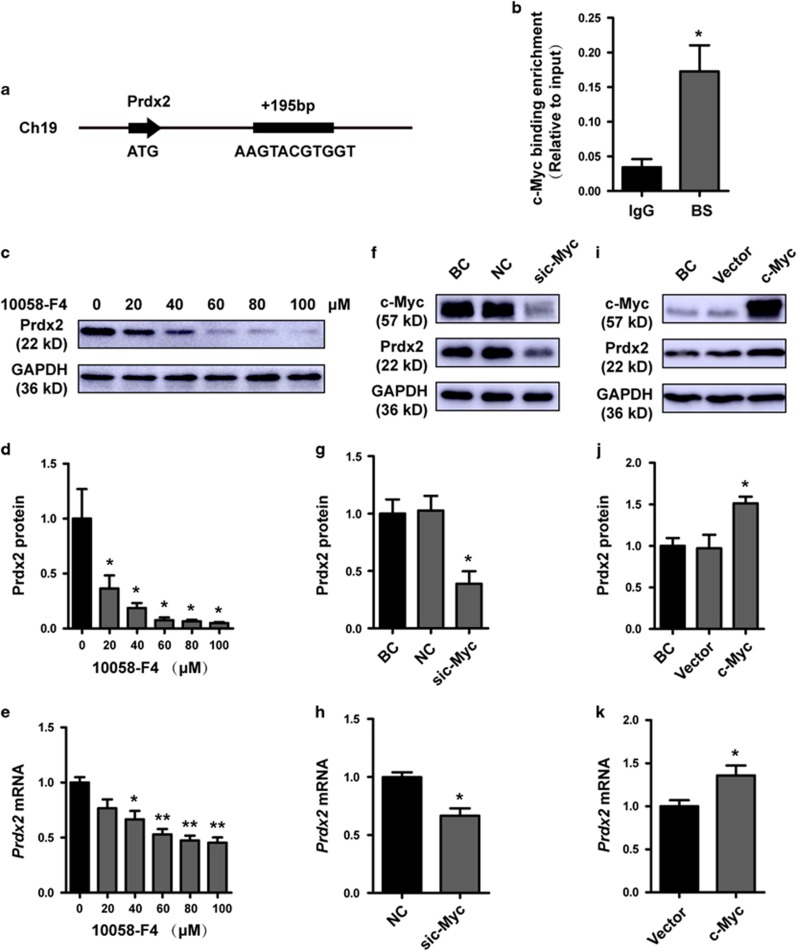
Prdx2 is regulated by c-Myc. (**a**) The diagram illustrates the c-Myc binding site (BS) in the promoter region of Prdx2. (**b**) ChIP assay was performed for immunoprecipitation of the potential c-Myc-binding regions of DNA. The DNA was then purified and amplified by quantitative RT-PCR. (**c**–**e**) Effect of the c-Myc-specific inhibitor 10058-F4 on the expression of Prdx2 in HTR8 cells was analyzed by western blot and quantitative RT-PCR analysis. (**f**–**k**) Western blot and quantitative RT-PCR analyses were used to analyze Prdx2 expression in HTR8 cells transfected with BC, siNC, sic-Myc, the control vector and the c-Myc vector. Data are expressed as the mean±S.E.M. *P<0.05 *versus* BC and NC, **P<0.001 *versus* BC and NC

We tried to identify the transcription factor that regulates Prdx2 expression by using the TRANSFAC tool. We found a c-Myc-binding site located in a region (+195 kb) present downstream of the Prdx2 initiation codon (Figure 7a). Chromatin immunoprecipitation (ChIP) was performed in HTR8 cells to determine whether c-Myc binds to this genomic locus. The results showed that c-Myc binds directly to the identified region of Prdx2 (Figure 7b). c-Myc needs to dimerize with its partner protein Max, a helix-loop-helix leucine zipper protein, to bind specific DNA sequences called E-box in the promoters of its target genes.^24^ Therefore, we used the c-Myc inhibitor 10058-F4, a small molecule that binds c-Myc monomers and disrupts c-Myc/Max dimerization, to interrupt the transcription function of c-Myc.^25^ We found that Prdx2 expression decreased with increase in the concentration of 10058-F4 (Figures 7c–e). c-Myc knockdown also decreased Prdx2 expression (Figures 7f–h), while overexpression of c-Myc increased the expression of Prdx2 in HTR8 cells (Figures 7i–k). These results indicate that c-Myc is a transcriptional activator that may directly mediate the expression of Prdx2.

### Downregulation of c-Myc in cytotrophoblasts from patients with RM and inhibition of cell proliferation and induction of cell apoptosis after c-Myc knockdown

**Figure 8:**
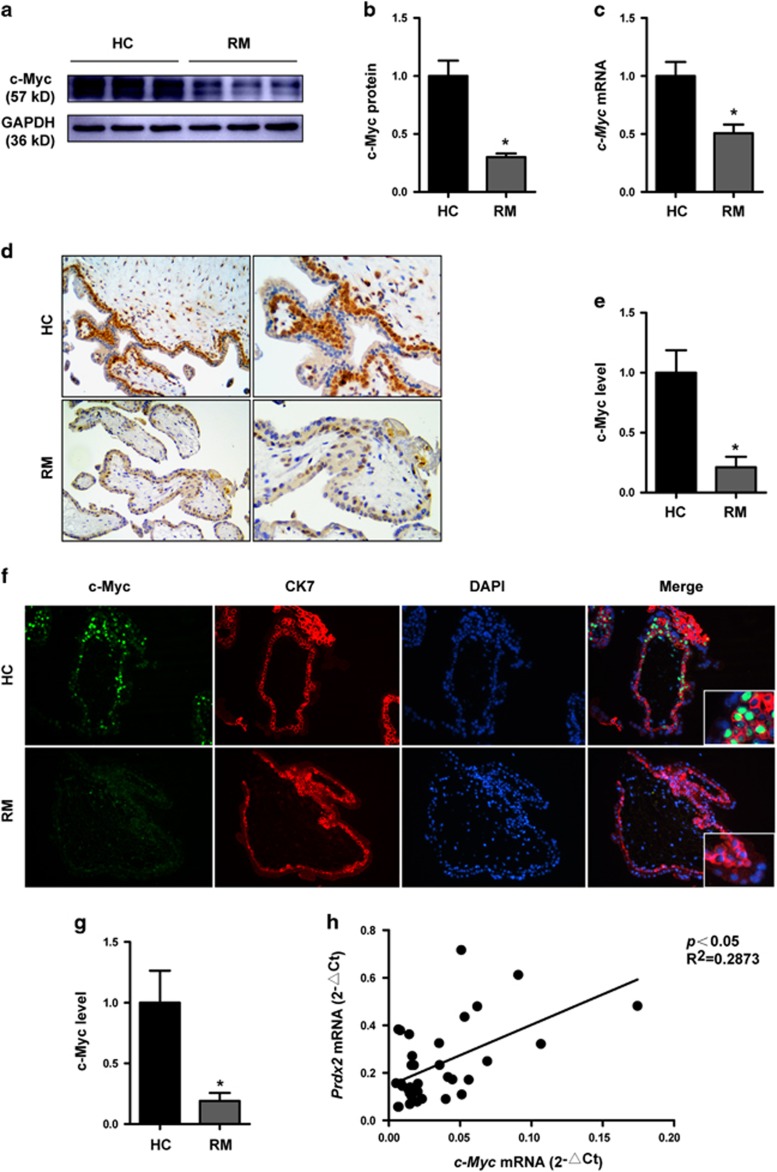

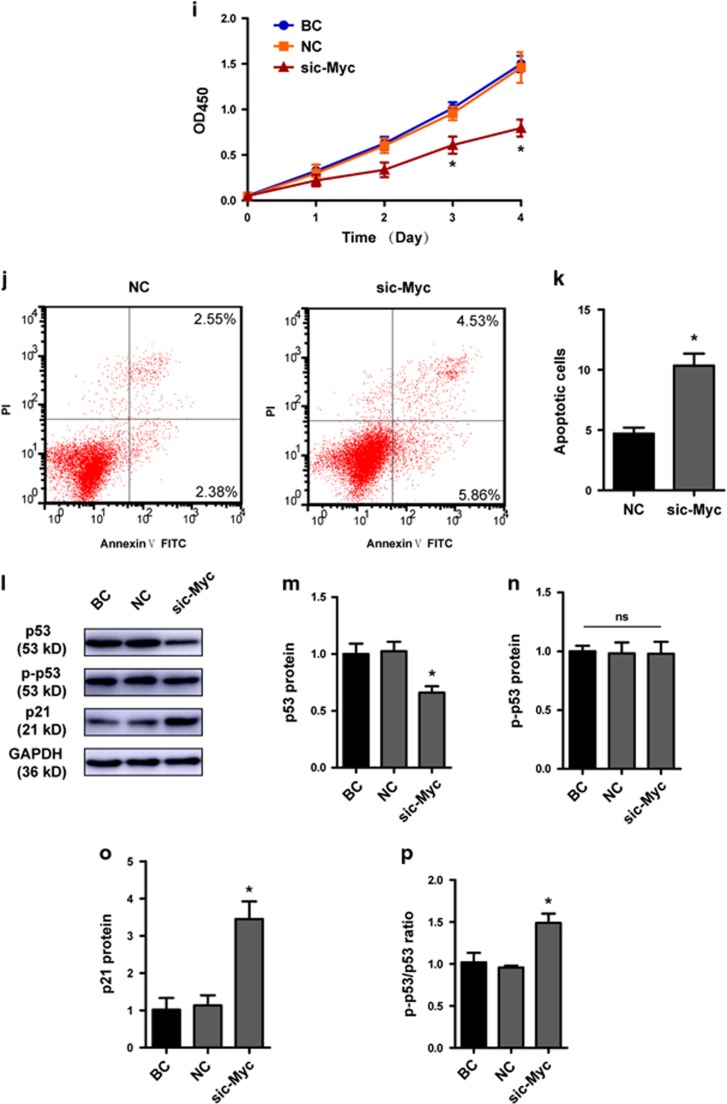
c-Myc expression is downregulated in first-trimester cytotrophoblasts in patients with RM and its knockdown inhibited cell proliferation and induced cell apoptosis. (**a**–**c**) c-Myc levels in first-trimester villous tissues from patients with RM and HCs were determined by western blot and quantitative RT-PCR analyses. The gray value of western blot bands was determined by Gel-Pro Analyzer 4.0. **b** and **c**: *n*=8 for HCs and *n*=10 for the RM group. (**d**,**e**) Immunohistochemical staining of c-Myc in sections of first-trimester maternal villi was performed using horseradish peroxidase-conjugated streptavidin–biotin complex with the chromogen 3,3-diaminobenzidine and counterstained with hematoxylin. *n*=12 for each group. Original magnification: 100 × (left), 200 × (right). (**f,g**) Double immunofluorescence staining for trophoblast marker CK7 with c-Myc was performed in first-trimester human villi from patients with RM and HCs. Green fluorescent signals represent c-Myc antibodies, red signals represent CK7 antibodies and the blue fluorescent signals represent nuclei. Images were obtained using a fluorescence microscope (Leica DMi8). *n*=11 for each group. Original magnification: 200 ×. Positive cells from both immunohistochemical and immunofluorescence analyses were quantified using ImagePro-plus 6.0. (**h**) The c-Myc and Prdx2 mRNA expression level in the villous tissue of patients with RM and HCs was determined using quantitative RT-PCR (*n*=36). Prdx2 mRNA expression was correlated with c-Myc mRNA expression in villous tissue. (**i**) Cell proliferation was analyzed by the CCK8 assay after HTR8 cells were transfected with sic-Myc and control siRNA. (**j**,**k**) The apoptosis rate of HTR8 cells was determined by flow cytometry analysis after cells were treated with control siRNA and sic-Myc. (**l**–**p**) The expression of p53, p-p53 and p21 were analyzed by western blot in control and sic-Myc transfected HTR8 cells. The gray value of western blot bands was determined by Gel-Pro Analyzer 4.0. Data are expressed as the mean±S.E.M. ns: no significance, *P<0.05 *versus* HCs, BC and NC

We examined the expression level of c-Myc in villous tissue from patients with RM and HCs. Consistent with the downregulation of Prdx2, c-Myc expression was also significantly decreased in the villi of the RM group (Figures 8a–c). IHC analysis of first-trimester villous tissue showed that c-Myc-positive cells were mainly found in the cytotrophoblast layer, and a much stronger positive signal was observed in HCs (Figures 8d and e). Double IF staining for trophoblast marker CK7 with c-Myc showed higher percentage of c-Myc^+^/CK7^+^ cells and stronger c-Myc staining signal in the HC group compared with RM group (Figures 8f and g). Linear correlation analysis showed that the Prdx2 mRNA level was correlated with the c-Myc mRNA level in villous tissue from the RM and HC groups (Figure 8h). These results indicate that both c-Myc and Prdx2 downregulation may be associated with the pathogenesis of RM. We knocked down c-Myc expression in HTR8 cells. The CCK8 assay showed that c-Myc knockdown decreased HTR8 cell proliferation (Figure 8i). We also observed increased HTR8 cell apoptosis rate after c-Myc knockdown by flow cytometry after annexin-V/propidium iodide staining (Figures 8j and k). Consistent with the effect of Prdx2, c-Myc downregulation increased the expression of p21. However, p53 expression was reduced with no change of the level of p-p53, thus the p-p53/p53 ratio was also upregulated after c-Myc knockdown (Figures 8l–p).

## Discussion

**Figure 9:**
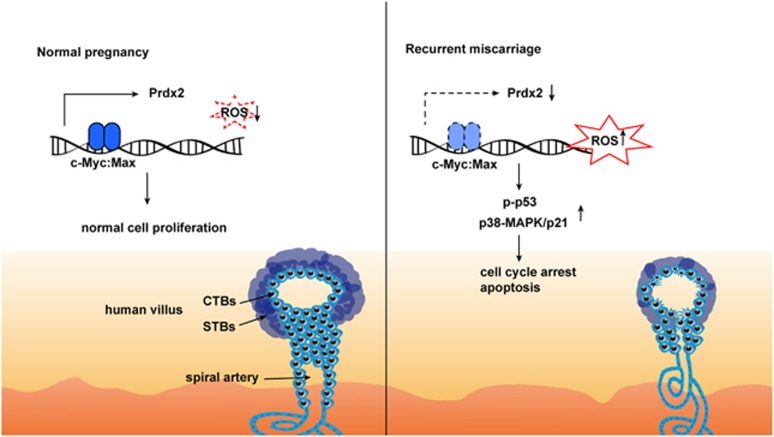
Diagrammatic representation of the study results. c-Myc regulates expression of the Prdx2 gene, which restricts the production of ROS and maintains normal trophoblast proliferation. Downregulation of Prdx2 is related with increase in trophoblast apoptosis and arrest of proliferation through ROS-related p-p53 and p38-MAPK/p21. The syncytiotrophoblast formation could also be damaged by Prdx2 downregulation. Whether the extravillous trophoblast (EVT) formation is related with Prdx2 is not known, although our previous research showed decreased outgrowth of EVTs from villi explants of RM patients compared with healthy controls.^28^ Prdx2 and its regulation by c-Myc may provide new insight into the molecular mechanism of RM, and these two proteins may be potential therapeutic targets. CTBs: cytotrophoblasts, STBs: syncytiotrophoblasts

Our study provides evidence for the biological function of Prdx2 in trophoblast proliferation and apoptosis and its potential role in RM (Figure 9).

In the present study, lower expression of Prdx2 was observed in first-trimester cytotrophoblasts obtained from RM patients. Further, *in vitro* Prdx2 knockdown weakened cell proliferation, diminished DNA synthesis and increased apoptosis in the first-trimester human extravillous cytotrophoblast-derived cell line HTR8. Villous cytotrophoblasts are progenitor cells that have strong proliferative ability and can fuse to form syncytiotrophoblasts or differentiate into extravillous trophoblasts.^3, 26, 27^ Normal cytotrophoblast function is important for implantation, spiral artery remodeling and maternal–fetal communication.^3, 26, 28^ Abnormal first-trimester trophoblast development and apoptosis is not only related with RM, but is also involved in many other pregnancy-related conditions such as preeclampsia and intrauterine growth restriction.^1^ Thus, Prdx2-targeted methods may open a new avenue for the diagnosis and treatment of dysfunction of early trimester trophoblasts in patients with RM. Further, Prdx2 may also have a role in other pregnancy-related disorders, which could be the subject of future investigations.

Our present findings showed that Prdx2 knockdown decreased the cell proliferation rate and increased the cell apoptosis rate of HTR8 cells. Consistent with our report, other study also demonstrated the existence of a close relationship between Prdx2 expression and cell cycle progression.^29^ For example, Prdx1- and Prdx2-knockout embryos were reported to exhibit a decrease in local proliferation, which led to aberrant morphogenesis.^30^ Cell cycle progression and apoptosis are closely linked activities.^31, 32, 33^ Some studies report that Prdx2 negatively regulates apoptosis-related signal-regulating kinase 1 (ASK1) activation by maintaining the reduction status of thioredoxin and inhibiting its dissociation from ASK1, thereby preventing activation of the downstream JNK and p38 pro-apoptosis pathways.^31, 32, 33^ Prdx2 also prevents ROS damage through the action of nuclear factor *κ*B (NF-*κ*B) and caspases.^34, 35^ Thus, Prdx2 may have potential as a therapeutic target in ROS-induced decrease in proliferation and increase in apoptosis.^36^

In this study, transfection of HTR8 cells with siRNA against Prdx2 resulted in an increase in the level of ROS and increase in the expression of p21 and p-p53/p53 ratio. Further, a higher apoptotic rate and higher expression of p-p53 and p21 were found in the villous tissues of patients with RM. The p53 expression was also upregulated in the villi of RM, while the reason may be related to other mechanisms. Phosphorylation can protect p53 from degradation by MDM2 and reinforce its activity to induce p21 expression.^37^ Both p-p53 and p21 have a damage-monitoring role in the genome that can inhibit cyclin-dependent kinase activity and cause cell cycle arrest or apoptosis in response to ROS attack.^5, 6, 38^ The underlying mechanism how Prdx2 activates p-p53 and p21 through ROS would require more in-depth investigation. According to other researches, ROS can induce DNA double strand breaks, which will activate ataxia-telangiectasia mutated (ATM) kinase.^39^ ATM can phosphorylate more than 700 substrates that are participated in plentiful cellular processes that include cell cycle control, DNA repair, transcription, apoptosis and proliferation.^39, 40^ The phosphorylation of p53 is one of the downstream activities that represent a damage repair mechanism to cause cell cycle arrest or apoptosis.^39, 40^

SB202190 is a selective inhibitor of p38 MAP kinase which specially inhibits the p38*α* and *β* isoforms, while SP600125 is a novel and selective inhibitor of JNK.^41, 42, 43^ These two kinases are closely implicated in the regulation of apoptosis, inflammation, and cell cycle progression thus their functions always overlap with each other.^42^ The different effects of SB202190 and SP600125 offer a solution to distinguish between p38-MAPK and JNK activated signal events.^42^ We found that p38-MAPK could be involved in Prdx2 knockdown-induced p21 expression. The underlying mechanism of these molecules and kinases in RM should be further explored, as they may represent potential therapeutic targets in pregnancy-related conditions. Increasing ROS may alter protein structure and function by oxidizing critical amino acid residues of MAPK signaling proteins to induce or mediate MAPK pathways.^44^ For example, ROS could induce oxidative modifications of receptor tyrosine kinases and MAP3Ks or inactivation of MAPK phosphatases, although the precise mechanisms are not known.^44^

In the present study, we found that decrease in cell viability and increase in the p-p53 and p21 levels could be rescued by the antioxidant NAC. Many antioxidants have been investigated for their potential effects in ameliorating ROS-related pregnancy disorders.^3^ However, the findings of many trials were not promising, and the exact effects of antioxidants in these disorders are still unclear.^3^ Thus, more investigations are required to explore the role of antioxidants in clinical practice.

Placental syncytiotrophoblast is responsible for the transport of oxygen, nutrients and wastes. It also produces hormones for fetal development and maintains immune tolerance.^26^ E-cadherin dynamically changes during the process of cytotrophoblast fusion and that its downregulation is coincident with cell fusion.^45^ Our results showed that Prdx2 knockdown could damage the BeWo cell fusion and decrease the expression of *Syncytin-2* induced by FSK. One research has demonstrated that the failure of differentiation and fusion into syncytiotrophoblast might be associated with an alteration in cellular redox state related to downregulated antioxidants.^46^ The downregulation of Prdx2 might promote apoptosis of cultured syncytiotrophoblasts or the reduced Prdx2 might influence the signal pathways that is involved in syncytialization.^14, 47^ The exact reason is lack of evidences and need further exploration.

Our study found that the transcription factor c-Myc targets the Prdx2 promoter and regulates Prdx2 expression. In addition, previous research has shown that the expression of Prdx2 can be regulated by FOXO3a and NF-*κ*B.^48, 49^ Analysis of human villous tissue in our study showed that RM patients had lower c-Myc levels and that c-Myc was correlated with Prdx2 expression. We predicted and verified the presence of only one c-Myc-binding site in the Prdx2 promoter: it was located downstream of the initiation site. There might have other putative c-Myc target regions and Prdx2 might also be directly or indirectly regulated by other redox-related transcription factors. However, its regulation by c-Myc may have a role in the pathogenesis of RM.

c-Myc is expressed ubiquitously during embryogenesis and carcinogenesis, and it can overcome cell cycle arrest and promote cell growth and proliferation.^24^ Our research proved that c-Myc downregulation inhibited cell proliferation, increased apoptosis and the expression of p21, which was consistent with the effect of Prdx2. Although the level of p53 was downregulated after c-Myc knockdown, p-p53 expression was not influenced, thus the percentage of p-p53 was also upregulated. Other reports have shown that c-Myc is capable of repressing p21^CIP1^ directly or through Ras-ERK-p21^CIP1^ to induce proliferation and p21^WAF1/CIP1^ expression was remarkably upregulated when c-Myc was knocked down.^50, 51, 52^ It has also been shown that c-Myc antagonizes the function of p53 and p53-induced p21^WAF1^ expression.^53^ The blocked expression of p53 by c-Myc inhibition was also found in other research and the reason may be due to post-translational regulation via increased MDM2 expression.^54, 55^ However, the percentage of p-p53 in total p53 was upregulated after c-Myc knockdown in our research. The reason might be related with the downstream Prdx2 repression and disturbed redox state. Inhibition of c-Myc induces cellular crisis through the synergistic effect of telomerase dysfunction and increase in oxidative stress.^56^ Other data also show that c-Myc downregulation lowers the apoptotic threshold.^57^

In summary, this study provides new insight into the pathogenesis of RM by demonstrating the role of Prdx2, which was found to regulate proliferation and apoptosis in early pregnancy through its effects on ROS metabolism. We also showed that Prdx2 expression was regulated by c-Myc, and that c-Myc and Prdx2 downstream effect might be related. Thus, downregulation of Prdx2 and c-Myc may be involved in the pathogenesis of RM, and therefore, therapeutic methods that target these two factors may be effective for the treatment of RM.

## Materials and methods

### Patient characteristics

Twenty-five patients with RM aged between 25 and 37 years of age were included in this study. All of them had been treated at the Department of Obstetrics and Gynecology of the International Peace Maternity & Child Health Hospital of the China Welfare Institute, Shanghai Jiao Tong University School of Medicine, China, between February 2015 and February 2016. All the patients have no uterine or cervical abnormality and no endocrine or metabolic diseases. Karyotype analysis of the parents or abortus showed that it was normal. Thirty-three women between the age of 21 and 35 years who underwent artificial abortion to terminate normal unwanted pregnancies at 6–12 weeks of gestation were recruited as HCs. All these women had experienced pregnancies without spontaneous abortion, preterm labor, or preeclampsia. The study was approved by the Medical Ethics Committee of the International Peace Maternity & Child Health Hospital of the China Welfare Institute, Shanghai. Written informed consent was obtained from all the participants before enrollment.

### Cell culture

The HTR8/SVneo cell line, which was derived from first-trimester human extravillous cytotrophoblasts, was a kind gift from Dr. PK Lala (University of Western Ontario, London, Ontario, Canada).^58^ The cells were cultured in Dulbecco’s modified Eagle’s medium/F12 containing 10% fetal bovine serum (Gibco, Grand Island, NY, USA) at 37 °C in a 5% CO_2_ atmosphere. BeWo cells, acquired from China Infrastructure of Cell Line Resources, Beijing, China, were cultured at 37 °C with 5% CO_2_ in Dulbecco’s modified Eagle’s medium/F12 containing 15% fetal bovine serum (Gibco).

### siRNA, plasmids and transfection

Two siRNAs targeting the human Prdx2 gene were designed: siPrdx2-1 (5′-GGAAGTACGTGGTCCTCTT-3′) and siPrdx2-2 (5′-GCCAGATCACTGTTAATGA-3′). The two siRNAs and a control siRNA were synthesized by Sangon Inc. (Shanghai, China). The siRNAs were transfected into HTR8 cells at a final concentration of 100 nmol/l using the Oligofectamine reagent (Invitrogen, Life Technologies, Carlsbad, CA, USA) according to the manufacturer’s protocol. Knockdown of c-Myc was performed using specific oligonucleotides purchased from Santa Cruz Biotechnology Inc. (TX, USA), which were transfected into cells using the Oligofectamine reagent (Invitrogen). All cells were cultured for 48 h after transfection before they were harvested for quantitative RT-PCR, western blot and other analyses. To create the c-Myc overexpression construct, the coding region sequence of human c-Myc was cloned into the pEX-2 vector (GenePharma, Shanghai, China). The c-Myc-pEX-2 vector and control vector were purified using the PureYield Plasmid Miniprep System (Promega, Madison, WI, USA), and transfected into the cells using Lipofactamine 3000 (Invitrogen) according to the manufacturer’s protocol. All cells were cultured for 48 h after transfection before other assays were performed.

### Quantitative RT-PCR

RNA was extracted from cells and human villous tissues using the TRIzol reagent (Life Technologies, Grand Island, NY, USA). The concentration and purity of the total RNA were assessed using a spectrophotometer (NanoDrop 2000c, Thermofisher Scientific, Waltham, MA, USA). cDNA was generated using a PrimeScript RT reagent kit with gDNA Eraser (perfect real-time) (Takara Bio, Kusatsu, Shiga, Japan), and quantitative PCR was performed using an SYBR Premix Ex Taq (Tli RNaseH Plus) (Takara Bio). The target PCR Ct values were normalized by subtracting the GAPDH Ct value. The relative expression level was calculated as follows: relative gene expression=2^−^^(△Ct sample^^−△Ct control)^. The following primers were used: GAPDH: 5′-TCAAGGCTGAGAACGGGAAG-3′ (forward) and 5′-TGGACTCCACGACGTACTCA-3′ (reverse), c-Myc: 5′-CGTCCTCGGATTCTCTGCTC-3′ and 5′-GCTGGTGCATTTTCGGTTGT-3′ (reverse), Prdx2: 5′-GTCCTTCGCCAGATCACTGT-3′ (forward) and 5′-GTCACTGCCAGGCTTCCA-3′ (reverse), Syncytin-2: 5′-GCAGCTCGTTTTGTGACCAG-3′ (forward) and 5′-CCGCCTCTATGCTTGTCCAT-3′ (reverse).

### Western blot analysis

The concentration of the protein extracts was calculated using a Pierce BCA protein assay kit (Pierce Biotechnology, Waltham, MA, USA) according to the manufacturer’s instructions. The proteins were separated using SDS-PAGE and transferred onto polyvinylidene difluoride membranes. The membranes were blocked for 1 h at room temperature using 5% non-fat milk. The membranes were then incubated with antibodies specific to Prdx2 (Abcam, Cambridge, UK), c-Myc (D3N8F; Cell Signaling Technology, Danvers, MA, USA), p21^waf1/cip1^ (12D1, Cell Signaling Technology), p53 (Cell Signaling Technology), p-p53 (Ser15, Cell Signaling Technology) and GAPDH (Abcam) overnight at 4 °C. After washing with Tris-buffered saline-Tween, the membranes were incubated with the corresponding secondary antibodies for 1 h. The membranes were then visualized using the Pro-light HRP chemiluminescent kit (Tiangen Biotech, Beijing, China) according to the manufacturer’s instructions and a chemiluminescence detection system (Amersham imager 600; GE Healthcare Life Sciences, Pittsburgh, PA, USA).

### Immunohistochemistry

Staining was performed using the immunohistochemical SABC kit (Boster, Wuhan, China) according to the manufacturer’s instructions and standard protocol.^59^ Paraffin-embedded sections were de-waxed and rehydrated, and antigen retrieval was performed by heating in a microwave oven in citrate buffer. The slides were then incubated in 3% hydrogen peroxide and blocked with 5% bovine serum albumin (BSA). After incubation with Prdx2 (Abcam), c-Myc (9E10, Santa Cruz, TX, USA), p21^waf1/cip1^ (Cell Signaling Technology), p53 (Cell Signaling Technology), p53 (phospho S15, Abcam), Ki67 (Cell Signaling Technology) and cleaved caspase-3 (Cell Signaling Technology) antibodies overnight at 4 °C, the slides were washed with phosphate-buffered saline (PBS) and incubated with secondary antibodies. After the slides were washed with PBS again, they were incubated with horseradish peroxidase-conjugated SABC and then the chromogen 3,3-diaminobenzidine and counterstained with hematoxylin. The slides were examined under a Leica DMi8 microscope (Leica Microsystems, Wetzlar, Germany) at 100 × and 200 × magnification.

### Explant culture

Explant culture was performed as described previously.^60^ Briefly, first-trimester healthy human placental villi were dissected into 2–3 mm tissues, explanted in 24-well culture plates precoated with phenol red-free Matrigel substrate (Corning, NY, USA), and cultured in DMEM/F12 medium with 10% FBS. To investigate the effect of Prdx2 on the proliferation in placental villi, eleven placentas were used. Explants from each placenta were divided into three groups, one for control siRNA treatment, two for siPrdx2-1 and siPrdx2-2 treatment, respectively.

Whole mount immunofluorescent staining was performed as described previously.^60^ Briefly, the explants cultured for 72 h together with matrigel were fixed by 4% paraformaldehyde. After blocking and permeabilization, the explants were incubated with primary antibodies against CK7 (Abcam and Cell Signaling Technology), Ki67 (Cell Signaling Technology) or Prdx2 (Abcam) at 4 °C for 24 h and fluorescent secondary antibody (Alexa Fluor 488-conjugated goat anti-Rabbit IgG, Alexa Fluor 594-conjugated goat anti-Mouse IgG) for 24 h. Finally, they were counterstained with Fluoroshield mounting medium with DAPI (Abcam) and then photographed using a fluorescence microscope (Leica DMi8 microscope, Leica Microsystems).

### Immunofluorescence analysis

Villous tissue slides were de-waxed, rehydrated and blocked with 5% BSA. After BSA was aspirated, the sections were incubated with primary antibodies against CK7 (Abcam), Prdx2 (Abcam) and c-Myc (Cell Signaling Technology) overnight at 4 °C. After the sections were washed with PBS, they were incubated with secondary fluorescent antibodies for 1 h at room temperature in the dark (Alexa Fluor 488-conjugated goat anti-Rabbit IgG and Alexa Fluor 594-conjugated goat anti-Mouse IgG, Life Technology). The slides were finally counterstained with Fluoroshield mounting medium with DAPI (Abcam) and then observed using a fluorescence microscope (Leica DMi8 microscope, Leica Microsystems).

For BeWo cells, coverslips were fixed with 4% paraformaldehyde, permeabilized with 0.5% Triton X-100, and blocked with 5% BSA. Cells were incubated with primary antibodies against E-cadherin (Cell Signaling Technology) overnight at 4 °C and fluorescent staining was performed using Alexa Fluor 594-conjugated goat anti-Rabbit IgG (Life Technology). Finally, cells were counterstained with Fluoroshield mounting medium with DAPI (Abcam). Fusion ratio was calculated as the number of nuclei in syncytial /total number of nuclei.

### Cell proliferation assay

HTR8 cells were plated at a density of 2 × 10^3^ cells per well in 96-well plates for the proliferation assay. Cell viability was analyzed at 24, 48, 72 or 96 h using the CCK8 assay (Dojindo, Kumamoto, Japan). For the NAC (Sigma-Aldrich, St. Louis, MO, USA) experiments, cells were treated with different concentrations of NAC (0–5 mM) for 24 h in 96-well plates before the CCK8 assay was performed. Absorbance was measured at 450 nm using a Synergy H1 microplate reader (BioTek, VT, USA).

### Flow cytometry analysis

An FITC Annexin-V Apoptosis Detection Kit with propidium iodide (Biolegend, San Diego, CA, USA) was used to detect apoptotic activity, according to the manufacturer’s instructions.

### BrdU assay

Cells were incubated with 10 *μ*M BrdU (Roche, Penzberg, Germany) for 2 h at 37 °C. The cells were then fixed with 4% paraformaldehyde, and denaturation of cellular DNA was performed with hydrochloric acid. After blocking and permeabilization, the incorporated BrdU was detected using anti-BrdU primary (Abcam) and secondary antibodies (Alexa Fluor 594-conjugated goat anti-Rabbit IgG, Life Technology). Sections were visualized with Leica DMi8 microscope (Leica Microsystems).

### TUNEL assay

Sections were de-waxed, rehydrated, and permeabilized using standard protocols, and then incubated with the TUNEL reaction mixture containing TdT and fluorescein-dUTP (*In Situ* Cell Death Detection Kit, AP, Roche). TdT can catalyze the attachment of fluorescein-dUTP to the DNA fragment ends. The incorporated fluorescein was detected with an AP-conjugated anti-fluorescein antibody with the chromogen 5-bromo-4-chloro-3-indolyl phosphate/tetranitroblue tetrazolium chloride and counterstained with nuclear fast red.

### Intracellular ROS assay

Cells were transfected with control siRNA and siPrdx2 for 48 h, and the intracellular ROS level was assessed with the oxidation-sensitive fluorescent dye DCFH-DA (Sigma-Aldrich) according to the manufacturer’s instructions. Fluorescence intensity was measured using the Synergy H1 microplate reader (BioTek) at an excitation wavelength of 488 nm and an emission wavelength of 520 nm.

### Chromatin immunoprecipitation

ChIP was performed using a ChIP kit (Millipore, Billerica, MA, USA) and antibodies against c-Myc (Abcam) together with negative control, input control and positive antibody control according to the manufacturer’s protocol. DNA immunoprecipitated from the sonicated cell lysates was quantified by real-time PCR (Takara Bio). Primers used in ChIP assay: 5′-CTAACTCCTCTGTGTCCCTCCTC-3′ (forward), 5′-AGCTTGCGGAAGTCCTCTGCA-3′ (reverse).

### Statistical analysis

Data are expressed as the mean±S.E.M.^61^ All *in vitro* experiments were performed at least three times. The independent sample *t*-test was used to compare two groups. Multiple groups were analyzed by one-way ANOVA with *post hoc* Tukey’s test.^41^ Correlations were analyzed using Spearman’s rank correlation test. All *P*-values are two-sided, and *P*<0.05 was considered to indicate statistical significance.
