# Supplementary figures and images for: Role of peroxiredoxin2 downregulation in recurrent miscarriage through regulation of trophoblast proliferation and apoptosis
Source: Cell Death Dis. 2017 Jun 29;8(6):e2908–. doi: 10.1038/cddis.2017.301 (PMC5520946; doi:10.1038/cddis.2017.301)

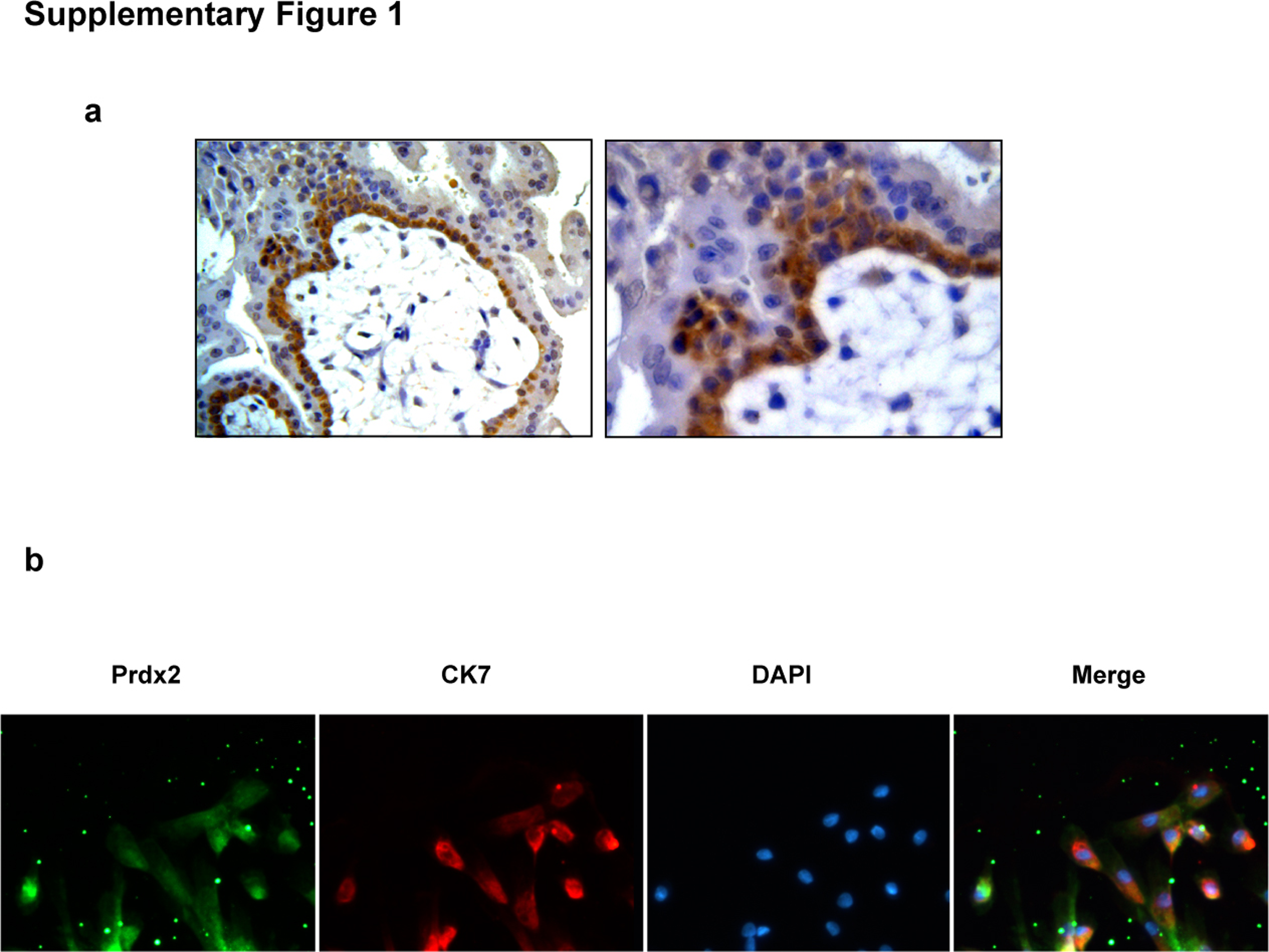

Supplement: Supplementary Figure 1 [file cddis2017301x1.tif]

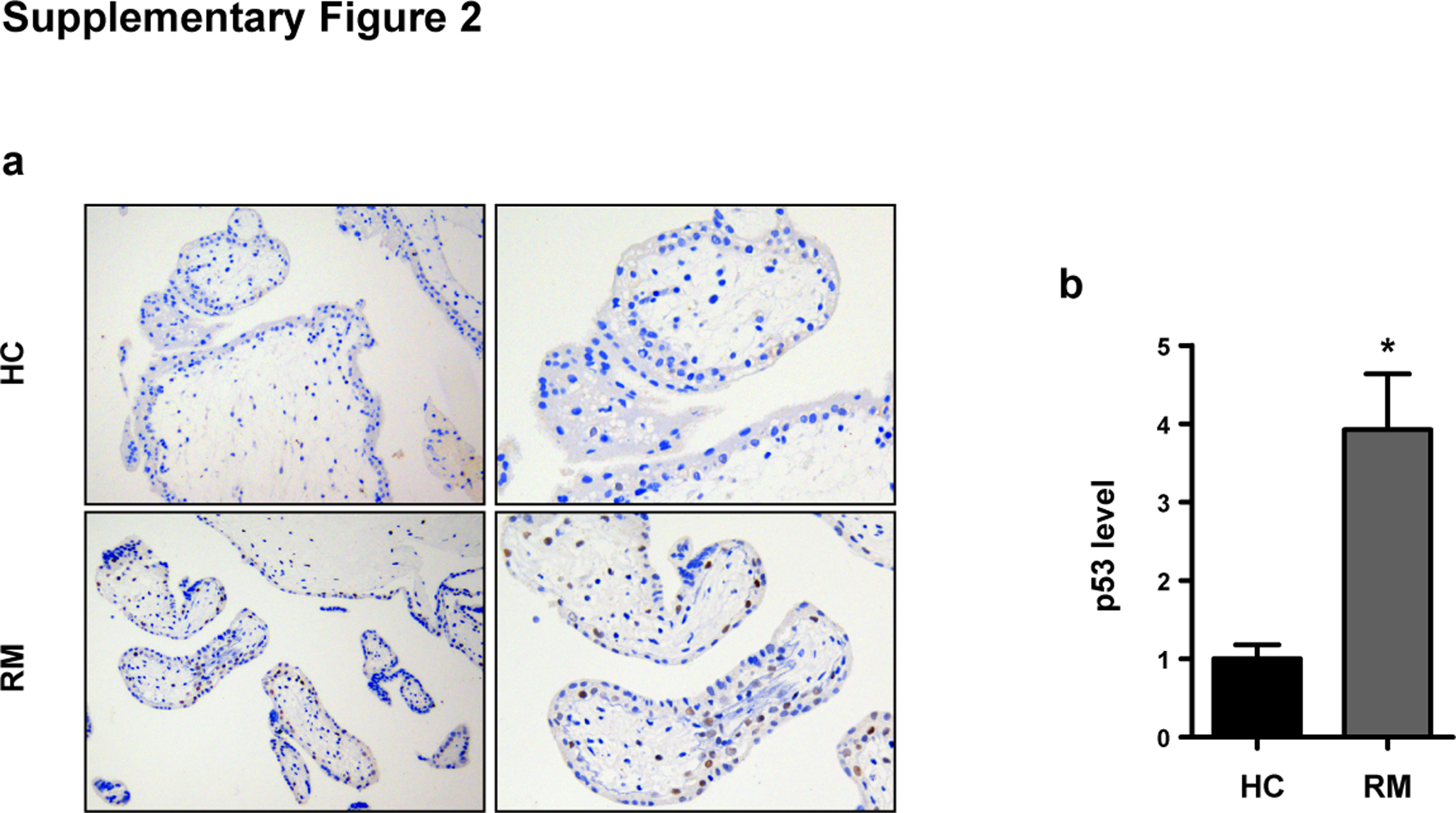

Supplement: Supplementary Figure 2 [file cddis2017301x2.tif]
