# Supplementary material for: Role of peroxiredoxin2 downregulation in recurrent miscarriage through regulation of trophoblast proliferation and apoptosis
Source: Cell Death Dis. 2017 Jun 29;8(6):e2908–. doi: 10.1038/cddis.2017.301 (PMC5520946; doi:10.1038/cddis.2017.301)
Supplement: Supplementary Figure Legends [file cddis2017301x3.doc]

**Supplementary Figure Legends**

**Supplementary Figure 1.** The trophoblastic cell columns and extravillous trophoblast cells also expressed Prdx2. (a) Immunohistochemical staining of Prdx2 in sections of healthy first-trimester villi with trophoblastic cell columns was performed using horseradish peroxidase-conjugated streptavidin–biotin complex with the chromogen 3,3-diaminobenzidine and counterstained with hematoxylin. Original magnification: 200× (left), 400× (right). (b) Whole mount immunofluorescent assay was used to analyze the expression of Prdx2 in healthy villi explants. Green fluorescent signals indicate Prdx2, red fluorescent signals indicate CK7 and the blue fluorescent signals represent nuclei. Original magnification: 200×.

**Supplementary Figure 2.** The expression of p53 was also upregulated in the villi of patients with RM. (a and b) Immunohistochemical staining of p53 in sections of first-trimester maternal villi was performed using horseradish peroxidase-conjugated streptavidin–biotin complex with the chromogen 3,3-diaminobenzidine and counterstained with hematoxylin. n = 10 for each group. Original magnification: 100× (left), 200× (right). Data are expressed as the mean ± SEM. **p* < 0.05 versus HCs.
